# Biological, Psychological and Social Determinants of Postpartum Depression: An Umbrella Review of Systematic Reviews and Meta-Analyses

**DOI:** 10.1192/bjo.2026.11240

**Published:** 2026-06-30

**Authors:** Rabiatul Adawiyah Binte Nor Idham, Chidinma Ayadiuno, Joshua Fakulujo

**Affiliations:** 1 King’s College London, London, United Kingdom; 2 GKT School of Medical Education, King’s College London, London, United Kingdom

## Abstract

**Aims:**

Postpartum depression (PPD) affects 10–25% of women worldwide, contributing to maternal morbidity, infant developmental issues, and elevated suicide risk. While PPD is widely recognised as multidimensional within the biopsychosocial model, comprehensive syntheses that integrate biological, psychological, and social determinants are limited. This umbrella review aims to identify and classify the strength and certainty of biological, psychological and social risk factors associated with PPD.

**Methods:**

This PRISMA-compliant umbrella review was prospectively registered on PROSPERO (CRD420251241065). Medline, Embase, PsycINFO, Scopus were searched from inception to November 2025. Systematic reviews and meta-analyses examining risk factors for PPD were included. Two independent reviewers screened titles/abstracts and full texts, with a third resolving discrepancies. Methodological quality was assessed using AMSTAR-2. Credibility was evaluated via umbrella review criteria (URC: Class I [convincing] to IV [weak]), and certainty using GRADE (high to very low).

**Results:**

Seventy-seven systematic reviews with meta-analyses, comprising 1658 studies and 25,737,929 women were included. Studies varied in quality and certainty. Biological factors associated with increased PPD risk included gestational diabetes mellitus (pooled OR/RR 1.3–2.7), caesarean section (OR 1.26–1.48), preterm birth/low birth weight (OR 1.79–1.97), perinatal pain (OR 1.29–1.75), and sleep disorders (OR 2.36–3.69). Psychological factors included antenatal depression (OR 4.58–7.70) and anxiety (OR 2.64–7.07), history of depression (OR 3.09–4.82), adverse childhood experiences (OR 2.31), and prenatal stress (PR 1.82). Social factors included intimate partner violence (OR 2.5–4.4), unintended pregnancy (OR 1.53–3.46), lack of social/family support (OR 2.57–5.96), and poor marital relationship (OR 3.47–3.56).

**Conclusion:**

Across biopsychosocial domains, obstetric complications (biological), antenatal mental health (psychological), and intimate partner violence (social) emerge as potential risks. High heterogeneity reflects variability in PPD definitions, timing, measurement tools and populations. Reliance on observational data precludes causality. Future research should focus on identifying and clarifying these risk factors to target screening and early intervention for at-risk mothers in varied contexts. Clinically, these findings support multidisciplinary interventions to mitigate PPD burden and improve maternal-infant outcomes.

